# Behavioural, psychiatric, and cognitive phenotypes associated with numbers of repeats of the FRAXE allele on the FMR2 gene

**DOI:** 10.12688/wellcomeopenres.21305.1

**Published:** 2024-05-08

**Authors:** Jean Golding, Marcus E. Pembrey, Rosie Clark, Yasmin Iles-Caven, Steven Gregory, Susan M. Ring, Sarah Ennis, Matthew Suderman

**Affiliations:** 1 Centre for Academic Child Health, Population Health Sciences, Bristol Medical School,, University of Bristol, Bristol, England, BS8 2BN, UK; 2 MRC Integrative Epidemiology Unit, Population Health Sciences, Bristol Medical School, University of Bristol, Bristol, England, BS8 2BN, UK; 3 Human Genetics and Genomics Medicine, University of Southampton, Southampton, SO16 6YD, UK

**Keywords:** ALSPAC, FRAXE, FMR2, psychosis, substance addiction, smoking, neurocognition, eating disorders

## Abstract

**Background:**

The FRAXE site on the X-chromosome has a variable number of trinucleotide repeats. The rare condition Fragile XE has >200 repeats, but most X chromosomes have <60 such repeats, with evidence of a bimodal distribution. It is known that when the number of repeats is <60, the repeat number can increase from mother to son, which raises the question as to whether there is an evolutionary advantage in the size of these repeats. This paper investigates whether the higher of the <60 repeats are associated with neurocognitive differences among boys in a general population. We hypothesised that although there was previous evidence of a link between higher numbers of repeats in the boys in this population with maternal grandmothers with schizophrenia, there may be cognitive or behavioural advantages to their grandsons of increased levels of repeats.

**Methods:**

We compared 1951 behavioural, psychiatric, and cognitive outcomes of 5060 boys from the Avon Longitudinal Study of Parents and Children (ALSPAC) using a phenome scan.

**Results:**

We found that boys with relatively high levels of repeats (>24) had a higher risk of certain neurocognitive outcomes (P<0.01). Boys with >24 repeats were more likely to report: (a) psychosis-like experiences; (b) increased ability to recognise facial signs of anger; (c) increased risk of eating disorders; (d) increased likelihood of smoking cigarettes and using illicit drugs during adolescence than would be expected by chance. There was no sign of associations with cognitive abilities.

**Conclusions:**

We concluded that there was little evidence that higher levels of the normal range of FRAXE repeats were associated with a difference in cognitive abilities, but there was evidence of increased reports of psychotic-like experiences and other behaviour problems in this group. There was no evidence of evolutionary neurocognitive advantage.

## Plain language summary

*Overview:* A specific area (FRAXE) on the X chromosome has a variable number of repeats of a GCC DNA sequence. Some adverse effects on boys’ cognitive ability are seen if there are 200+ repeats but less is known about cognitive/other effects associated with lower repeat numbers which rarely exceed 60. We examined neurocognitive and behavioural outcomes associated with <60 repeats of FRAXE and considered 1951 outcomes concerning behaviour, psychiatric disorders, temperament and cognitive abilities. For each outcome we determined whether it was a linear association (i.e. whether higher repeat numbers increased the risk of that outcome) and whether boys with 24–60 repeats had a greater risk than boys with <25 repeats. Analysis used 5060 boys participating in ALSPAC (Avon Longitudinal Study of Parents and Children), followed-up throughout childhood via structured self-completion questionnaires by mothers, fathers, teachers and the children themselves; testing within a clinical setting; linkage to national health and education records. Comparison of boys with >24 repeats with those with <25 repeats revealed more significant associations than expected by chance involving psychotic experiences, increased ability to identify facial anger expressions, eating disorders and addictive behaviours. Each association exhibited linear relationships (the higher the number of repeats the more likely the boy to have the outcome).

*Why is it important?* Evidence exists indicating numbers of FRAXE repeats on the mother’s X-chromosome can increase when that chromosome is inherited by her child. If this confers an evolutionary advantage, identifying the mechanism regarding which beneficial outcome may be responsible could be important.

*Key takeaway:* We concluded that higher numbers of repeats was unlikely to confer evolutionary advantages due to male neurocognitive outcomes, however, we suggest that other attributes of increased repeat numbers may be of evolutionary importance.

## Introduction

The FRAXE section of the FMR2 gene, located on the X chromosome [ Knight *et al.*, 1993], contains varying numbers of trinucleotide repeats. As we noted previously [ Clark *et al.*, 2020], studies comparing the number of repeats between mother and son have shown that there can be changes in the number, but this instability only occurs at levels of repeat approaching 40 or more; from that point onwards there is evidence that the rate of instability increases with the greater the number of repeats [ Murray *et al.*, 2000]. This change over time begs the question as to whether there is an evolutionary advantage in increasing numbers of repeats. Boys with over 200 repeats (when the gene is methylated) tend to have mild cognitive impairments, but little is known concerning the phenotypes of individuals with smaller numbers of repeats. The full mutation has an estimated prevalence of 1 in 23,400 males [ Crawford *et al.*, 1999]. In most cases the full mutation of FRAXE results in milder cognitive impairment than for the FRAXA phenotype (Fragile X), which is the expansion of the CGG repeat in the FMR1 gene [ Crawford *et al.*, 1999]. There are examples in the literature of males with a full mutation at FRAXE who have no cognitive impairment [ Crawford *et al.*, 1999; Gecz *et al.*, 1997], but there is sometimes evidence of behavioural problems, reading and writing difficulties, obsessive compulsive disturbance, autistic behaviour, hyperactivity, poor adaptive skills, anxiety and aggression [ Dennis *et al.*, 1992; Hamel *et al.*, 1994; Knight *et al.*, 1996]. There has also been some suggestion in the literature that the Xq28 locus, the AFF2 gene and FRAXE full mutation are associated with schizophrenia [ Au-Young & Gould 2001; Hosak *et al.*, 2012; Nelson & Gu, 2006; Sullivan *et al.*, 2008].

In this paper we describe the results of a phenome scan of neurocognitive outcomes associated with the number of FRAXE repeats in the male offspring (known as the G1s) from the Avon Longitudinal Study of Parents and Children (ALSPAC), utilising the vast array of data collected from questionnaires, interviews and examinations over time. An earlier study has shown that the mothers of these boys (who will almost certainly have had a similar number of repeats on one of their X chromosomes) were marginally less likely than their peers to have achieved a higher education or high occupation level in adulthood. Interestingly, we have shown that these maternal grandmothers were more likely to have schizophrenia if the X chromosome of their grandsons had a relatively high number (>21) of FRAXE repeats (OR 4.81; 95% CI 1.70, 13.6; P = 0.003) [ Golding *et al.*, 2021]. Thus, there is some evidence of a link between FRAXE repeats and schizophrenia.

The present study has fine detail on a relatively large number of neurocognitive outcomes which have been derived from the actual answers to specific tests or questions (traits), although reported diagnoses are also included. We have approached the outcome information using a phenome scan approach by determining associations with neurocognitive outcomes across the age range from 6 months to 16 years. We assess both whether the group of boys with the highest level of repeats differs from the rest of the population, and whether there is a trend of increasing (or decreasing) risk with increasing numbers of FRAXE repeats. The aims are to determine whether there are outcomes that would indicate whether there were signs of evolutionary advantages to having relatively large numbers of repeats.

## Methods

### The ALSPAC sample

Pregnant women resident in Avon, UK with expected dates of delivery from 1st April 1991 to 31st December 1992 were invited to take part in the study. The initial number of pregnancies enrolled was 14,541 (for these at least one questionnaire has been returned or a “Children in Focus” clinic had been attended by 19/07/99). Of these initial pregnancies, there was a total of 14,676 fetuses, resulting in 14,062 live births and 13,988 children who were alive at 1 year of age, 7353 of whom were boys. The overall aim of the ALSPAC study was to determine ways in which the environment (in association with the genotype) was associated with the health and development of the child [ Boyd *et al.*, 2013; Fraser *et al.*, 2013; Golding *et al.*, 2001].

The majority of the environmental data was collected from self-completion questionnaires completed by the mothers, their partners, and later by the child and the child’s teachers. Data included psychosocial background, physical environments, and health of the parents. Also collected were details of the child’s cognitive and motor development, temperament and behaviour over time. These data included validated structured tests using maternal self-completion questionnaires, tests administered by ALSPAC staff under standardised conditions, and national academic test results.

Further details of the study methodology, enrolment and response rates are available on the study website ( http://bristol.ac.uk/alspac/index.html). The study website also contains details of all the available data through a fully searchable data dictionary and variable search tool ( http://www.bristol.ac.uk/alspac/researchers/our-data/).

### DNA amplification and analysis

The venepuncture blood samples obtained in ALSPAC clinics from children at 43 and/or 61 months were stored for one month to two years before DNA extraction, and samples taken at seven years were stored for up to three weeks. The cord blood samples which were collected at birth were stored in heparin at -70 ^o^C for five to eight years before DNA extraction [ Jones *et al.*, 2000]. DNA was forwarded from those for whom there was a sufficient blood sample and appropriate consent for genetic research to the Wessex Regional Genetics Laboratory where they were prepared for FRAXE analysis as described below [ Ennis *et al.*, 2006].

The DNA was amplified using PCR with fluorescently labelled oligonucleotide primers across the GCC FRAXE repeat. Further details of the PCR reaction are given elsewhere [ Ennis *et al.*, 2006]. The data were then analysed on 672 GENESCAN software (ABI/Perkin Elmer) and imported into GENOTYPER software (ABI/Perkin Elmer) to assign alleles [ Murray *et al.*, 1996]. The technique used could not identify the children with the full mutation – although with a prevalence of 1 in 23,000 this was unlikely to have meant the omission of more than one case.

**Figure 1.**
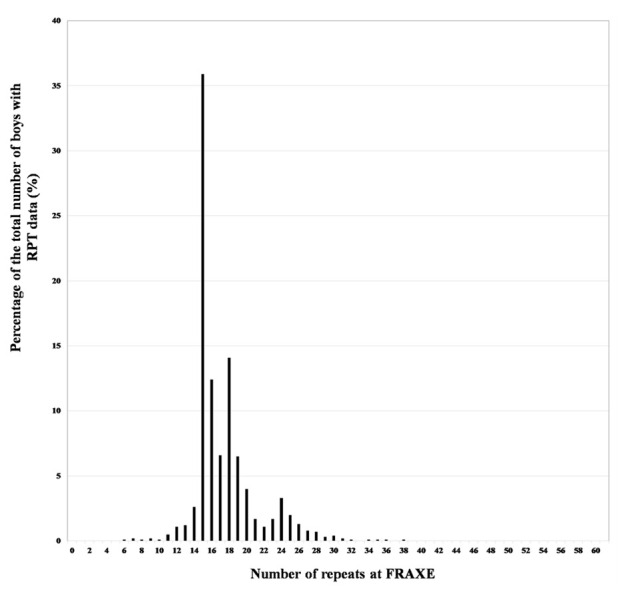
Distribution of the FRAXE repeats in the ALSPAC boys. [Reproduced from: Clark R, Gregory S, Ring S *et al.* The FRAXA and FRAXE allele repeat size of boys from the Avon Longitudinal Study of Parents and Children (ALSPAC) [version 2; peer review: 2 approved]. *Wellcome Open Res* 2020, 4:116 ( https://doi.org/10.12688/wellcomeopenres.15342.2)]

The frequency of the various numbers of repeats in this population is shown in Figure 1 where there is evidence for the distribution to be described as approximately bimodal, with modes at 15 and 24 repeats. This distribution was identical to that found with a different population by Murray *et al*. [2000].

### The phenomes

 Various measures have been collected over time concerning the study child. These have included: (a) specific questions to the child’s carer, to the primary school teacher and to the child him/herself; (b) direct testing and observation of the child; and (c) linkage to results of national examinations. Aspects of each of these measures have been used to create sets of phenomes: biological, sensory, neurocognitive, and specific health conditions. Each set is being accumulated over time and is identified by the date on which it was completed.

The current study concerns the set of phenomes compiled on 12 ^th^ February 2020. It comprises 1860 outcome measures divided into six sections:

- Cognition: comprising measures of early child development, IQ, memory, speech and language, reading ability, test results on spelling, mathematics and science, executive function, reasoning, non-verbal communication and musicality.
- Temperament and behaviour: early measures of child temperament, sleeping behaviour, feeding behaviour, temper tantrums, general childhood behaviour over time, selective attention, unusual behaviours observed by ALSPAC staff, and gender type behaviour.
- Personality: locus of control, self-esteem, cognitive style, personality, borderline personality disorder, antisocial behaviour, sensation seeking, friendships and bullying, and perception of own abilities.
- Motor abilities: laterality, balance, and motor coordination.
- Traits commonly found in psychiatric disorders such as depression, psychosis, eating disorders, and self-harm.
- Sources of addiction: behaviours involving alcohol consumption, cigarette smoking, cannabis use, other illicit drug use, and gambling.

### Statistical analyses

This hypothesis generator is deliberately a simplistic set of analyses and is best described as a search for pattern. It first analyses the data using two different methods: (a) using logistic regression to compare children with relatively high (>24) repeats (24 being the mode of the second peak of the distribution) with those with lower numbers of repeats; and (b) using linear multiple regression to compare the relationship between the mean number of repeats and the various outcome measures. Where both methods show similar results, we assume that there is evidence of an association.

For each set of phenomes, the numbers of associations observed at different P values were compared with the numbers that would be expected by chance. Where there were significantly more observed phenotype associations than expected (i.e. ([obs – exp] ^2^ / exp) was greater than 3.84), the component phenotypes making up these results were investigated in more detail. This format is similar to the strategy we have also used when analysing other phenome scans [e.g. Golding *et al.*, 2020].

## Results

In all there were 7573 boys enrolled in the ALSPAC study of whom 5060 (66.8%)had a measure of the FRAXE repeats of sufficient quality for statistical analysis [ Clark *et al.*, 2020].

### Bias in availability of FRAXE repeats

The maternal background of the boys enrolled within ALSPAC for whom there are measures of FRAXE repeats available are shown in Table 1. This indicates that mothers for whom their sons’ FRAXE repeat levels were available were more likely to be older, working in non-manual occupations and living in homes that they either owned outright or had mortgaged. However, there was no difference between the first or later born boys in the likelihood of their FRAXE repeats being determined.

**Table 1.** The proportion (no.) of boys involved in the ALSPAC study for whom a measure of FRAXE was available.

Demographic measure	Proportion (n) with FRAXE available
*Maternal age at birth of her son*	
<20	46.4% (150)
20–24	56.9% (778)
25–29	67.7% (1879)
30–34	70.1% (1387)
35+	72.5% (547)
*Parity*	
1 ^st^ born	67.5% (1983)
2 ^nd^ born	67.8% (1588)
3 ^rd^ + born	68.2% (933)
*Social class*	
I and II	72.1% (1283)
III Nonmanual	70.7% (1713)
Manual	63.9% (951)
*Housing tenure*	
Owner occupied	71.0% (3501)
Other	57.5% (1030)

### Associations with the phenotypes

 As demonstrated in Table 2a and Table 2b, the boys’ numbers of FRAXE repeats were linked to their 1951 neurocognitive phenotypes and assessed for: (i) binary associations between <25 and >24 repeats, and (ii) the overall linear trend with number of repeats. Of the 1951 phenotypes tested, 122 binary and 121 trend analyses showed associations at P<0.05, more than the 97.6 expected (P<0.05) ( Table 2a), and 29 and 38 of the tests were associated at P<0.01 compared with 19.5 expected ( Table 2b). Thus, the data indicate that there were overall more associations at P<0.05 than expected. Below we describe the six subgroups of phenotypes in more detail.

**Table 2a.** The numbers of items/traits tested, the numbers of tests expected to differ by chance at P<0.05 and the numbers observed at P<0.05, comparing (i) those with >24 repeats with the rest, (ii) the trend in mean no. of FRAXE repeats across the variables in each of six neurocognitive domains. (*observed associations differ from expected (P<0.05)).

Domain	No. items Tested	No. expected	No. observed >24 v.<25	No. observed Linear trend
Cognition	525	26.3	41 *	20
Temperament and behaviour	636	31.8	22	21
Personality	271	13.6	12	24 *
Motor	102	5.1	5	1
Psychiatric disorders	248	12.4	17	33 *
Addictions	169	8.5	25 *	22 *
ALL OUTCOMES	1951	97.6	122 *	121 *

**Table 2b.** The numbers of items/traits tested, the numbers of tests expected to differ by chance at P<0.01 and the numbers observed at P<0.05, comparing (i) those with >24 repeats with the rest, (ii) the trend in mean no. of FRAXE repeats across the variables in each of six neurocognitive domains. (*observed associations differ from expected (P<0.05)).

Domain	No. items Tested	No. expected	No. observed >24 v.<25	Linear trend
Cognition	525	5.3	6	5
Temperament And behaviour	636	6.4	2	5
Personality	271	2.7	3	7 *
Motor	102	1.0	0	0
Psychiatric disorders	248	2.5	8 *	13 *
Addictions	169	1.7	10 *	8 *
ALL OUTCOMES	1951	19.5	29 *	38 *

### Measures of cognition

 Altogether 525 measures of cognition were examined covering topics such as IQ, speech, and language, reading and other scholastic ability levels, reasoning, and non-verbal communication. By definition, 26.3 and 5.3 would be expected at P<0.05 and P<0.01 respectively; the actual numbers observed were 41 and 6 of the >24 repeat comparisons, and 20 and 5 of the linear trend analyses ( Table 3 and Table 4). Thus, the only indication of more associations at P<0.05 than expected occurred in the binary comparisons and not in the linear trend analyses.

**Table 3.** The number of variables tested against FRAXE repeats, the numbers observed and the numbers expected comparing >24 with <25 repeats according to P value. (*observed associations differ from expected (P<0.05)).

Topic	No. items	P<0.05		P<0.01		P<0.001	
		ob.	exp	ob.	exp	ob.	exp
*Cognition*							
Early child development (<5y)	46	2	2.3	0	0.5	0	0.1
IQ (8y; 15y)	24	0	1.2	0	0.2	0	<0.1
Memory (8–17y)	28	0	1.4	0	0.2	0	<0.1
Speech and language	185	17 *	9.3	2	1.4	0	0.1
Reading ability	77	6	3.9	2	0.8	1	0.1
Other test results	71	7	3.6	0	0.7	0	0.1
Executive function	29	2	1.5	1	0.3	0	<0.1
Reasoning	10	3	0.5	0	0.1	0	<0.1
Non-verbal communication	41	4	2.1	1	0.6	1	<0.1
Musicality	14	0	0.7	0	0.1	0	<0.1
ALL COGNITION	525	41 *	26.3	6	5.3	2 *	0.5
*Temperament and behaviour*							
Temperament	62	0	3.1	0	0.6	0	<0.1
Sleep behaviours	127	12 *	6.4	2	1.3	0	0.1
Feeding behaviours	100	1	5.0	0	1.0	0	0.1
Temper tantrums	14	0	0.7	0	0.1	0	<0.1
SDQ behaviours	84	2	4.2	0	0.8	0	0.1
DAWBA behaviours	129	2	6.5	0	1.3	0	0.1
DAWBA diagnoses	45	1	2.4	0	0.5	0	<0.1
Selective attention	28	0	1.4	0	0.3	0	<0.1
Unusual behaviour observed	38	1	1.8	0	0.4	0	<0.1
Gender behaviour	9	3 *	0.5	0	0.1	0	<0.1
ALL TEMPERAMENT AND BEHAVIOUR	636	22	31.8	2	6.4	0	0.6
*Personality*							
Locus of control	21	0	1.1	0	0.2	0	<0.1
Self-esteem	78	3	3.9	1	0.8	0	0.1
Child’s perception of ability	50	0	2.5	0	0.5	0	<0.1
Cognitive style	24	4 *	1.2	1	0.2	0	<0.1
Personality	10	1	0.5	0	0.1	0	<0.1
Borderline personality disorder	12	0	0.6	0	0.1	0	<0.1
Antisocial behaviour	38	0	1.9	0	0.4	0	<0.1
Sensation seeking	5	0	0.3	0	0.1	0	<0.1
Friendships and bullying	33	4	1.7	1	0.3	0	<0.1
ALL PERSONALITY	271	12	13.6	3	2.7	0	0.3
*Motor ability*							
Laterality	25	2	1.3	0	0.3	0	<0.1
Balance	59	3	2.9	0	0.6	0	0.1
Manual abilities	18	0	0.9	0	0.2	0	<0.1
ALL MOTOR ABILITIES	102	5	5.1	0	1.0	0	0.1
*Traits of Psychiatric Disorders*							
Depression	77	0	3.9	0	0.8	0	0.1
Psychosis	30	4 *	1.5	3 *	0.3	1	<0.1
Eating disorders	84	9 *	4.2	3 *	0.8	1	<0.1
Self-harm	57	4	2.9	2	0.6	0	0.1
ALL TRAITS	248	17	12.4	8 *	2.5	2	0.2
*Sources of Addiction*							
Alcohol consumption	55	3	2.8	2	0.6	1	0.1
Cigarette smoking	20	11 *	1.0	5 *	0.2	0	<0.1
Cannabis	33	4	1.7	0	0.3	0	<0.1
Other illicit drug	39	6 *	1.9	3 *	0.4	0	<0.1
Gambling	22	1	1.1	0	0.2	0	<0.1
ALL ADDICTIVE BEHAVIOURS	169	25 *	8.5	10 *	1.7	1	0.2
TOTAL OUTCOMES *	1951	122 *	97.6	29 *	19.5	5 *	2.0

**Table 4.** The number of variables tested with trends in mean levels of FRAXE repeats, the numbers observed and the numbers expected according to P value. ( *observed associations differ from expected (P<0.05)).

Topic	No. items	P<0.05		P<0.01		P<0.001	
		ob.	exp	ob.	exp	ob.	exp
*Cognition*							
Early child development (<5y)	46	3	2.3	2 *	0.5	0	0.1
IQ (8y; 15y)	24	3	1.2	0	0.2	0	<0.1
Memory (8–17y)	28	1	1.4	0	0.2	0	<0.1
Speech and language	185	2 *	9.3	0	1.4	0	0.1
Reading ability	77	3	3.9	0	0.8	0	0.1
Other test results	71	0	3.6	0	0.7	0	0.1
Executive function	29	1	1.5	0	0.3	0	<0.1
Reasoning	10	1	0.5	0	0.1	0	<0.1
Non-verbal communication	41	5 *	2.1	3 *	0.6	3 *	<0.1
Musicality	14	1	0.7	0	0.1	0	<0.1
ALL COGNITION	525	20	26.3	5	5.3	3 *	0.5
*Temperament and behaviour*							
Temperament	62	2	3.1	1	0.6	0	<0.1
Sleep behaviours	127	4	6.4	1	1.3	0	0.1
Feeding behaviours	100	4	5.0	1	1.0	0	0.1
Temper tantrums	14	0	0.7	0	0.1	0	<0.1
SDQ behaviours	84	3	4.2	0	0.8	0	0.1
DAWBA behaviours	129	1 *	6.5	1	1.3	0	0.1
DAWBA diagnoses	45	3	2.4	1	0.5	0	<0.1
Selective attention	28	0	1.4	0	0.3	0	<0.1
Unusual behaviour observed	38	1	1.8	0	0.4	0	<0.1
Gender behaviour	9	3 *	0.5	0	0.1	0	<0.1
ALL TEMPERAMENT AND BEHAVIOUR	636	21	31.8	5	6.4	0	0.6
*Personality*							
Locus of control	21	3	1.1	0	0.2	0	<0.1
Self-esteem	78	8 *	3.9	4 *	0.8	0	0.1
Child’s perception of ability	50	7 *	2.5	2 *	0.5	0	<0.1
Cognitive style	24	1	1.2	0	0.2	0	<0.1
Personality	10	1	0.5	1	0.1	0	<0.1
Borderline personality disorder	12	0	0.6	0	0.1	0	<0.1
Antisocial behaviour	38	4	1.9	0	0.4	0	<0.1
Sensation seeking	5	0	0.3	0	0.1	0	<0.1
Friendships and bullying	33	0	1.7	0	0.3	0	<0.1
ALL PERSONALITY	271	24 *	13.6	7 *	2.7	0	0.3
*Motor ability*							
Laterality	25	1	1.3	0	0.3	0	<0.1
Balance	59	0	2.9	0	0.6	0	0.1
Manual abilities	18	0	0.9	0	0.2	0	<0.1
ALL MOTOR ABILITIES	102	1	5.1	0	1.0	0	0.1
*Traits of Psychiatric Disorders*							
Depression	77	4	3.9	3 *	0.8	1	0.1
Psychosis	30	9 *	1.5	4 *	0.3	2 *	<0.1
Eating disorders	84	9 *	4.2	3 *	0.8	0	<0.1
Self-harm	57	11 *	2.9	3 *	0.6	0	0.1
ALL TRAITS	248	33 *	12.4	13 *	2.5	3 *	0.2
*Sources of Addiction*							
Alcohol consumption	55	4	2.8	2	0.6	0	0.1
Cigarette smoking	20	8 *	1.0	2 *	0.2	0	<0.1
Cannabis	33	4	1.7	0	0.3	0	<0.1
Other illicit drug	39	6 *	1.9	4 *	0.4	0	<0.1
Gambling	22	0	1.1	0	0.2	0	<0.1
ALL ADDICTIVE BEHAVIOUR	169	22 *	8.5	8 *	1.7	0	0.2
TOTAL OUTCOMES	1951	121 *	97.6	38 *	19.5	4	2.0

Splitting the cognition group into the various phenotypic sub-sections ( Table 3 and Table 4) showed that speech and language were associated at P<0.05 for both sets of analyses. For the group of >24 repeats there were more associations than expected, but most of these indicated better abilities in regard to speech and language such as vocabulary, grammar, and intelligibility. For the linear analysis, there were fewer associations than expected at P<0.05. There were very few associations at the P<0.001 level, but more than expected by chance – the excess was due to associations in the non-verbal communication section. The strongest of these concerned the DANVA series of facial expressions [ Nowicki & Duke, 2008] – affected boys were markedly more likely to identify angry faces as such (e.g. for the boys with >24 repeats, OR 3.33 [95%CI 1.56, 5.00]). This pattern was also true of the linear trend where this level of association was particularly apparent in relation to the high intensity angry expressions, such that the mean number of FRAXE repeats showed a trend - the more FRAXE repeats, the more likely was the boy to recognise extreme anger (P<0.0001). Similar trends were not obvious for recognition of other expressions such as happiness or sadness (data not shown).

### Temperament and behaviour

 Overall, 636 variables were assessed concerning temperament and behaviours of the child at various ages. Although 31.8 would have been expected by chance to demonstrate associations at P<0.05, there were fewer such associations observed (22 and 21 respectively for the two different analyses ( Table 3 and Table 4). Of the different components considered, there were more associations with sleeping behaviour than expected for the boys with >24 repeats (they were more likely to have difficulty going to sleep, to wake during the night and have nightmares), but there were no such increased findings with the linear trend analyses. For both sets of analyses there were more associations than expected with gender behaviour (observed 3, expected 0.5 for each set of analyses). The gender behaviour tests in children comprised details of the types of play that the boy preferred and has been used to create a score indicating the degree of masculine type behaviour at three different ages. The data indicated that the boys with higher numbers of repeats were more likely than those with fewer repeats to have a particularly typical male-type behaviour profile at the age of 8 [mean difference +2.13; 95%CI +0.46, +3.8] for those with >24 repeats].

### Personality

 Of the 271 measures assessed, no more than expected showed associations at P<0.05 when the comparisons were between >24 and less, but there were more associations than expected with the linear trend analyses ( Table 2a and Table 2b). The actual measures involved concerned the child’s perception of their own abilities whereby, in spite of these children being on a par with others in regard to their abilities when tested (as shown in the cognition section), they were more likely to report themselves as worse than others. Similarly, there were differences in the linear trend analyses with measures of lower self-esteem ( Table 4).

### Motor ability

 There were no more associations than expected between the 102 measures of motor coordination and balance compared with the numbers of FRAXE repeats.

### Psychiatric disorders

 The 248 measures of (mostly) components of psychiatric conditions did show an overall excess in the numbers observed that varied at P<0.05 and P<0.01, particularly when the linear trends with numbers of repeats were considered ( Table 2a and Table 2b). The differences were consistently contributed to by the indicators of possible psychosis and eating disorders. There was also an excess of associations that possibly indicate self-harm with the linear trend analyses ( Table 3 and Table 4).

 The indicators of possible psychosis comprised questions asked when the boys were approximately 16 years of age, using the Psychosis Like Symptoms (PLIKS) scale in two formats – sent within a self-completion questionnaire to the study children at their own home, and a face-to-face psychiatric interview within a clinic environment [ Niarchou *et al.*, 2015]. The self- completion questionnaire provided more positive answers, and therefore more statistical power. It was largely these questions that showed the associations shown in Table 3 and Table 4. The actual questions used are shown in relation to the linear trend in Table 5. It can be seen that even when the p value is >0.05, the data are consistent in showing increases, rather than decreases, in the number of repeats in the answers to those psychotic-like symptoms for which the boy states that he has definitely experienced them.

 For eating disorders, questions were asked on food avoidance in three questionnaires sent to the mother (or chief carer) when the adolescent was 13 years 1 month, 13 years 10 months and 16 years of age. The relevant questions are shown in Table 6 with the proportions of high repeats and the mean repeat numbers. The pattern indicated is of an increased risk of food avoidance with the higher number of repeats. For example, of the boys who, at age 16, felt that they had a strong desire for food ‘like an addiction’, 21.4% had >24 repeats compared with 5.7% who had no such feelings. The numbers are small in this high repeat group, but the associations are mirrored by comparison with the pattern shown when the mean FRAXE repeats are considered ( Table 6).

**Table 5.** The mean [and standard deviation] of the no. of FRAXE repeats of 16-year-olds according to their responses to questions on psychosis-like experiences in a self-completion questionnaire.

Experience	Definitely	Maybe	No	P
Believes his thoughts have been read	18.31[3.50]	17.24[3.99]	17.31[3.77]	0.454
Believes he has been followed or spied upon	17.34[4.68]	17.87[5.19]	17.28[3.56]	0.129
Heard voices others couldn’t hear	17.61[3.95]	17.64[6.02]	17.29[3.58]	0.339
Saw something no one else could see	19.59[7.72]	18.10[3.95]	17.23[3.58]	**<0.0001**
Felt thoughts were being taken out of his head against his will	23.33[8.02]	17.43[3.44]	17.28[3.80]	**0.022**
Felt someone else’s thoughts were inserted into his head against his will	22.60[6.27]	19.30[8.55]	17.23[3.61]	**<0.0001**
Felt his thoughts were so loud others could hear them	20.00[5.66]	18.07[4.53]	17.27[3.75]	**0.024**
		**Yes**	**No**	
Believes received special messages through radio or TV		18.65[6.72]	17.24[3.60]	**0.004**
Felt under the control of a special power		18.61[3.89]	17.30[3.78]	**0.028**
Felt they were really special or had special powers		17.88[5.60]	17.28[3.64]	0.131

**Table 6.** Proportion of boys who answered and had >24 repeats, and the mean [SD] no. of repeats according to the questions on feeding avoidance behaviours at ages 13 and 16.

Question	Age	%(n) >24 FRAXE	Mean [SD] FRAXE
*Avoids food that will make him fat*	13.1		
No		5.4 (113)	17.26 [3.58]
A little		7.5 (50)	17.69 [3.94]
A lot		9.5 (<5)	18.31 [3.64]
P		**0.028**	**0.004**
*Frequency avoids fattening foods*	13.1		
Never		5.0 (83)	17.19 [3.71]
Sometimes		7.3 (76)	17.65 [4.18]
Mostly		8.6 (8)	17.98 [4.47]
Always		-	17.44 [2.60]
P		**0.009**	**0.001**
*Eats less at mealtimes to avoid putting on weight*	16		
No		5.7 (111)	17.33 [3.94]
A little		4.8 (10)	17.12 [3.77]
A lot		23.1 (<5)	19.54 [4.59]
P		**0.044**	0.811
*Skipped meals to avoid putting on weight*	13.1		
No		5.7 (149)	17.33 [3.80]
Yes		10.8 (15)	17.99 [5.27]
P		**0.014**	**0.050**
*Refused to eat food that the mother thought they should*	13.1		
Often		9.2 (15)	17.62 [4.67]
Occasionally		6.1 (63)	17.32 [3.92]
Never		5.3 (84)	17.35 [3.76]
P		0.058	0.668
*Spends a lot of time thinking about food*	13.10		
Yes		8.3 (30)	17.80 [4.86]
No		5.6 (115)	17.25 [3.74]
P		**0.047**	**0.014**
*Has a strong desire for food like an addiction*	13.10		
Not at all		6.0 (153)	17.34 [3.86]
A little		7.9 (18)	17.82 [4.85]
A lot		8.0 (<5)	18.00 [4.16]
P		0.256	0.056
*Has a strong desire for food like an addiction*	16		
Not at all		5.7 (118)	17.33 [3.94]
A little		3.1 (<5)	17.17 [3.28]
A lot		21.4 (<5)	19.93 [5.03]
P		**0.044**	**0.042**
*Would be ashamed if others knew how much he ate*	16		
Yes		27.3 (<5)	18.82 [4.98]
No		5.7 (120)	17.33 [3.87]
P		**0.008**	0.203

### Exposure to factors that could result in addiction

There were 169 measures of use of substances (other than food) which could become the basis of addiction. These included alcohol, cigarettes, cannabis, other illegal drugs and sources of gambling. There were considerably more significant associations at both the 0.05 and 0.01 levels than would be expected by chance ( Table 2a and Table 2b).

These associations were predominantly due to cigarette smoking and the use of ‘other drugs’. The associations with cigarette smoking were consistent over time and identified that the boys with more repeats were more likely to start smoking earlier in adolescence, and to smoke more often ( Table 7). Although cannabis use was not particularly associated with the number of repeats, there were associations with taking drugs such as ecstasy, amphetamines and hallucinogens, with the users being more likely to have a higher number of FRAXE repeats ( Table 7).

**Table 7.** Proportion of boys (n) with >24 repeats, and the mean [SD] no. of repeats according to the questions on cigarette smoking and ‘other’ illicit drugs used.

Question	Age asked	%(n) >24 FRAXE	Mean [SD] FRAXE
*Cigarette smoking*			
*-age started*	18		
< 11		15.4 (6)	18.79 [5.50]
11–12		9.5 (9)	17.89 [4.15]
13–15		6.7 (18)	17.28 [4.18]
Not by 16		4.9 (27)	17.12 [3.46]
P		**0.004**	**0.005**
*No. smoked at 16*	16		
None		5.0 (71)	17.22 [3.67]
< 1/week		10.2 (10)	17.58 [3.87]
< 1/day		10.0 (<5)	17.55 [3.57]
1+/day		10.4 (11)	17.93 [4.66]
P		**0.004**	**0.041**
*Ever smoked*	18		
Yes		8.0 (33)	17.54 [4.29]
No		4.9 (27)	17.12 [3.46]
P		**0.048**	**0.036**
***Examples of ‘Other drugs’***			
*Used ecstasy*	16		
Yes		11.0 (10)	18.09 [4.33]
*No*		5.8 (90)	17.29 [3.74]
P		**0.046**	**0.049**
*Used amphetamines*	18		
Yes		10.7 (11)	18.21 [5.04]
No		5.3 (45)	17.14 [3.60]
P		**0.033**	**0.007**
*Used hallucinogens*	20		
Yes		11.6 (20)	18.24 [4.88]
No		5.8 (66)	17.30 [3.61]
P		**0.005**	**0.002**

## Discussion

This study is, as far as we know, unique in assessing the way in which the number of FRAXE repeats is associated with neurocognitive characteristics in a general population of boys studied throughout childhood and adolescence. Although there is evidence in the literature of individuals with the full mutation (>200 repeats) having relatively low IQ, we have found no associations with number of repeats among those without the full mutation, of any reduction in IQ or abilities in early development or in their competence in academic subjects, apart from improved abilities in their speech and language development (which did not reach P<0.05). Nevertheless, there were some signs of certain neurocognitive abnormalities during adolescence regarding increased likelihood of psychotic-like symptoms, eating behaviours, increased ability to recognise anger in facial expressions, use of cigarettes and illicit drugs.

The association found between boys with relatively large numbers of repeats at FRAXE and schizophrenic phenotypes is possibly related to reports in the literature concerning the Xq28 locus, the *AFF2* gene and FRAXE full mutation having associations with diagnosed schizophrenia [ Au-Young & Gould, 2001; Hosak *et al.*, 2012; Sullivan *et al.*, 2008; Nelson & Gu, 2006]. In addition, one study has identified 27 differently expressed genes in Fragile XE patients, two of which have an associated phenotype of schizophrenia [ Melko *et al.*, 2013]. These reports together with the finding from this cohort of a markedly increased risk of schizophrenia in the maternal grandparents of the boys with high levels of FRAXE repeats [ Golding *et al.*, 2021] adds credence to a possible causal effect (50% of the X chromosomes of the boys will have been inherited from their maternal grandmothers). Although the numbers were small (only 15 maternal grandmothers were known to be schizophrenic), six of them had grandsons with high levels of FRAXE repeats (OR 4.81 [95% CI 1.70, 13.6] P=0.003), and the mean number of repeats among the 15 exceeded that of the rest of the population (mean difference +3.43 [95%CI +1.43, +5.42] P<0.001).

There is little evidence in the literature to test our finding of an association of high numbers of repeats at FRAXE with early onset of cigarette smoking. However, some studies have shown that active smokers exhibit a higher frequency of fragile site expression in general, but that this is transient and reversible after cessation of smoking [ Stein *et al.*, 2002].

Fragile sites such as FRAXE that are prone to instability might be expected to be detrimental and therefore evolutionarily selected against [ Arlt *et al.*, 2006]. Yet the evolutionary conservation of common fragile sites is apparent; they persist in genomes of organisms ranging from yeast to humans [ Arlt *et al.*, 2006]. These sites may be just a consequence of the higher-order chromosome structure or the transcriptional regulation of associated genes [ Durkin & Glover, 2007]. Another theory is that the fragility, in itself, serves a valuable biological function; they are late replicating and could serve to signal to the cell that replication is complete [ Durkin & Glover, 2007]. Given this, it could even be possible that relatively high numbers of repeats at fragile sites such as FRAXE are evolutionarily advantageous. If this was a general feature of fragile sites, one might expect there to be strong correlations between the numbers of repeats at different fragile sites; however, there was only a very weak correlation between the numbers of repeats at the FRAXA and FRAXE sites (r = 0.027; data not shown). Nor were the numbers of FRAXA repeats associated with the phenotypes found here with FRAXE.

Our study has indicated some potential advantages to the individual of increased numbers of FRAXE repeats since they were associated with better development of verbal communication skills with improved speech and language, as well as increased and appropriate non-verbal skills restricted to awareness of anger in others; this may well provide a survival advantage [ Nowicki & Duke, 2013]. However, if true these associations may not be balanced by the adverse psychiatric characteristics in adolescence shown here.

These findings raise the question as to possible biological mechanisms. We have shown elsewhere evidence that FRAXE repeat length is strongly associated with **cis** DNAm in this non-clinical population and suggest that this may mediate the effect of repeat length on downstream phenotypes via gene expression [ Suderman, personal communication].

### Strengths and limitations

 The strengths of the study lie in the following: (a) it is nested within a population birth cohort; unlike family studies, this project is not contingent on the presence of a family member with Fragile XE. (b) Data were collected on the behaviour and development of the study children throughout childhood and adolescence using self-completion questionnaires completed at various time points by parents, teachers and the study child itself, blind to the FRAXE status of the children. (c) Assays to determine the number of FRAXE repeats were carried out without knowledge of the neurocognitive development of the study boys.

 Limitations concern: (i) Apart from the possible findings with schizophrenia we have not been able to find any evidence in the literature to confirm (or deny) our other findings in regard to FRAXE repeat size as no studies to our knowledge have looked in this regard at features such as eating disorders, self-esteem or use of substances prone to induce addiction. (ii) The methodology we have used may result in increased or reduced expected numbers as the assumption is that all the items considered are mutually independent (which they are not); however, we suggest that the results should be valid hypothesis raisers. (iii) Although the size of our study is larger than other studies of FRAXE repeats, it is very possible that many of the association tests carried out were underpowered.

## Conclusion

Using this population of child and adolescent boys we have found some evidence that those with an increased number of repeats at the FRAXE locus were more prone to certain disadvantageous neurocognitive traits including increased exposure to substances likely to be addictive, eating disorders and psychotic type experiences. Although our aim was to determine whether there was any clue as to whether the increase in the lengths of repeats over generations could be explained by some evolutionary advantage, we were unable to identify any such advantage in the cognitive and behaviour domains studied here. However, we cannot rule out beneficial effects on physical outcomes that may explain such an advantage in adult men or women.

 It has been assumed that although the full mutation at FRAXE may result in some cognitive abnormalities, lower numbers of repeats are unlikely to result in any such problems. By taking a broad-based approach we have shown consistent associations between signs indicative of psychosis/schizophrenia and the upper end of the distribution of repeats. It is important to test whether other signs shown here such as eating disorders and propensity to use addictive type substances are repeated in other datasets.

## Ethics and consent

Ethical approval for the study was obtained from the ALSPAC Ethics and Law Committee and the Local Research Ethics Committees [ Birmingham, 2018]. Questionnaires were completed in parents’ homes and then were returned to the study offices. Returning of the questionnaires was interpreted as giving tacit consent to involvement in the study. Members of the study always have the right to withdraw their consent for elements or the entirety of the study at any time. The biological samples were collected with participants’ consent in accordance with the Human Tissue Act [2004], and only analysed if the mother had given signed consent. Details of the approvals obtained are available in full from the ethics pages of the study website ( http://www.bristol.ac.uk/alspac/researchers/research-ethics/).

## Data Availability

ALSPAC data access is through a system of managed open access. The steps below highlight how to apply for access to the data included in this paper and all other ALSPAC data. Please read the ALSPAC access policy ( http://www.bristol.ac.uk/media-library/sites/alspac/documents/researchers/data-access/ALSPAC_Access_Policy.pdf) which describes the process of accessing the data and biological samples in detail, and outlines the costs associated with doing so. 1. You may also find it useful to browse our fully searchable research proposals database ( https://proposals.epi.bristol.ac.uk/), which lists all research projects that have been approved since April 2011. 2. Please submit your research proposal ( https://proposals.epi.bristol.ac.uk/) for consideration by the ALSPAC Executive Committee using the online process. You will receive a response within 10 working days to advise you whether your proposal has been approved. If you have any questions about accessing data, please email: alspac-data@bristol.ac.uk (data) or bbl-info@bristol.ac.uk (samples). The ALSPAC data management plan ( http://www.bristol.ac.uk/media-library/sites/alspac/documents/researchers/data-access/alspac-data-management-plan.pdf) describes in detail the policy regarding data sharing, which is through a system of managed open access.
