# Supplementary material for: Behavioural, psychiatric, and cognitive phenotypes associated with numbers of repeats of the FRAXE allele on the FMR2 gene
Source: Wellcome Open Res. 2025 Apr 30;9:238. Originally published 2024 May 8. [Version 2] doi: 10.12688/wellcomeopenres.21305.2 (PMC12287684; doi:10.12688/wellcomeopenres.21305.2)
Supplement: Peer review history

# Reviewer report: Reviewer response for version 2

**DOI:** 10.21956/wellcomeopenres.25480.r125809

**Published:** 2025-07-23

**Authors:** Kayla Smith

**Affiliations:** 1 University of Warwick, Coventry, England, UK

This manuscript compared the behavioural, psychiatric, and cognitive outcomes of boys from the ALSPAC using a phenome scan, finding that boys with relatively high levels of repeats (>24) had a higher risk of certain neuro-cognitive outcomes as well as increased reports of psychotic-like experiences and other behaviour problems. This manuscript presents a well-designed and clearly written study that addresses an important gap in the current literature as there is a dearth of research on mutations on the FRAXE section of the FMR2 gene. The methodology is rigorous, and the data are well-analysed. The results were presented logically and compellingly. The authors provided a thoughtful discussion, which contextualised the findings within the broader field and was supported by their results. This work makes a valuable contribution to the understanding of different phenotypes based on FRAXE repeats.

Is the work clearly and accurately presented and does it cite the current literature?

Yes

If applicable, is the statistical analysis and its interpretation appropriate?

Yes

Are all the source data underlying the results available to ensure full reproducibility?

Yes

Is the study design appropriate and is the work technically sound?

Yes

Are the conclusions drawn adequately supported by the results?

Yes

Are sufficient details of methods and analysis provided to allow replication by others?

Yes

Reviewer Expertise:

My area of research is primarily focused on fragile X syndrome and other rare genetic syndromes, with a focus on profiles of anxiety and autism as well as characterising behavioural, physiological, and cognitive phenotypes in these conditions.

I confirm that I have read this submission and believe that I have an appropriate level of expertise to confirm that it is of an acceptable scientific standard.

# Reviewer report: Reviewer response for version 2

**DOI:** 10.21956/wellcomeopenres.25480.r123577

**Published:** 2025-06-03

**Authors:** Chioma N. P. Mbachu

**Affiliations:** 1 Nnamdi Azikiwe University, Nnewi, Anambra, Nigeria

Thank you for inviting me to review this manuscript entitled  “Behavioural, psychiatric and cognitive phenotypes associated with numbers of repeats of the FRAXE allele on the FMR2 gene”

 General comments

 I commend the authors for utilising a large population-based dataset (ALSPAC), which has the potential to uncover new genotypic-phenotypic associations.

 However, the authors should consider these concerns.

 1.      The analytic sample (“FRAXE repeats of sufficient quality for statistical analysis”) appears to unevenly comprise participants from socioeconomically advantaged backgrounds (e.g., higher maternal age, higher income, higher education) and from a specific area of south-west regional health authority. The authors should discuss the implications sufficiently because this bias in selection could have introduced confounders  that affect associations particularly in behaviour and cognition, and affect generalisability.

 2. It is unclear if statistical corrections were done for this large number of outcomes (e.g Bonferroni). If yes, it should be clearly mentioned to rule out false positives.

Is the work clearly and accurately presented and does it cite the current literature?

Yes

If applicable, is the statistical analysis and its interpretation appropriate?

Partly

Are all the source data underlying the results available to ensure full reproducibility?

Yes

Is the study design appropriate and is the work technically sound?

Partly

Are the conclusions drawn adequately supported by the results?

Partly

Are sufficient details of methods and analysis provided to allow replication by others?

Yes

Reviewer Expertise:

Child and adolescent health, neurodevelopmental disorders, Fragile X syndrome, Fragile X Premutation-associated conditions

I confirm that I have read this submission and believe that I have an appropriate level of expertise to confirm that it is of an acceptable scientific standard, however I have significant reservations, as outlined above.

# Reviewer report: Reviewer response for version 1

**DOI:** 10.21956/wellcomeopenres.23565.r86731

**Published:** 2024-07-03

**Authors:** Marsha R Mailick

**Affiliations:** 1 Waisman Center, University of Wisconsin-Madison, Madison, Wisconsin, USA

This is a population-based study of the association between the number of GCC repeats in the FRAXE allele on the FMR2 gene and various phenotypic characteristics in boys. The data were drawn from the Avon Longitudinal Study of Parents and Children (ALSPAC), a birth cohort from Avon, UK that has collected an enormous amount of data about the children’s development across a broad array of outcomes as well as the health of their parents. The ALSPAC has been a resource used by studies seeking to link genetics and aspects of the environment with the health and development of children.

 There has been very limited prior study of phenotypic associations of the number of GCC repeats in the FRAXE allele, apart from the full mutation. The authors speculate about the possibility of an evolutionary advantage of the number of GCC repeats below the full mutation, which is an important question, though not directly addressed in the present report. There is some indication in the literature of a possible neurocognitive profile associated with varying number of GCC repeats and, in a previous report, of an association with psychiatric difficulties in earlier generations.

 The greatest strengths of the present study are the very large number of participants in ALSPAC (over 7,000 boys), and the fact that it is an unbiased population-based birth cohort. Moreover, participants were not aware of their genetic status, nor was their participation in ALSPAC motivated by a family history of disease. These strengths set the present study apart from other studies of genotype-phenotype correlations. It is also comprehensive, spanning multiple phenotypes as measured by 1,860 outcome measures categorized into six domains (e.g., cognition, temperament and behavior, etc.).

 The findings of the present study suggest that boys with greater than 24 repeats had a higher risk of certain neurocognitive outcomes, such as psychosis-like experiences, eating disorders, and certain addictive behaviors. The authors present this work as hypothesis generating, and a “deliberately simplistic set of analyses”.

 A limitation of the study is that there is an overwhelming amount of data, and data reduction approaches should be considered. For example, in the cognition domain, there are 10 separate measures and together they total 525 tested items. The analytic approach reported here attempts to deal with the massive amount of data by comparing the number of significant differences observed versus the number of differences expected, at p values of .05, .01. and .001. Adjusting for multiple comparisons within each of the six domains by a Bonferroni or some other method would greatly strengthen confidence in the phenotypic patterns that emerge.

 Very few studies have focused on phenotypic variations associated with repeat numbers below clinical thresholds, and the present study potentially would open the door to this type of investigation, especially if some approach to data reduction would be employed.

Is the work clearly and accurately presented and does it cite the current literature?

Yes

If applicable, is the statistical analysis and its interpretation appropriate?

Partly

Are all the source data underlying the results available to ensure full reproducibility?

Yes

Is the study design appropriate and is the work technically sound?

Partly

Are the conclusions drawn adequately supported by the results?

Partly

Are sufficient details of methods and analysis provided to allow replication by others?

Yes

Reviewer Expertise:

epidemiology of neurodevelopmental disorders

I confirm that I have read this submission and believe that I have an appropriate level of expertise to confirm that it is of an acceptable scientific standard, however I have significant reservations, as outlined above.

# Response

**Published:** 2024-09-16

**Authors:** Yasmin Iles-Caven

**Affiliations:** Bristol Medical School: Population Health Sciences, University of Bristol, Bristol, UK

**Thank you for the suggestion of using a Bonferroni type method to reduce the large number of associations. We have now pointed out in the paper the dangers of this approach in that one is likely to make both Type I and Type II errors. It is also important to note that such an approach fails to consider the likelihood of pleiotropic effects, which we have now emphasised. Ideally, these findings will in future be tested in other large populations.**

 Very few studies have focused on phenotypic variations associated with repeat numbers below clinical thresholds, and the present study potentially would open the door to this type of investigation, especially if some approach to data reduction would be employed.

**We did consider data reduction to avoid strong collinearity between traits. However, we decided against it as any observed associations would be difficult to interpret. We hope that our study will indeed open the door to similar studies in other populations in which replication of our findings can be investigated.**

# Reviewer report: Reviewer response for version 1

**DOI:** 10.21956/wellcomeopenres.23565.r83713

**Published:** 2024-05-30

**Authors:** Paul J Hagerman

**Affiliations:** 1 University of California, Davis, CA, 95616, USA

The authors presented an analysis of the relationship between the number of GCC repeats in the3 range <60 and various neurocognitive and behavioral phenotypes, largely developed through surveys. The authors had speculated that the apparent instability in mother-to-son transmission, with increasing GCC repeat number in the offspring, might reflect an evolutionary advantage. However, they saw no cognitive benefit, and noted that the larger repeats were associated with several disadvantageous neurocognitive traits. The results are intriguing, although the basis for the observed associations is not known. This study should provide a stimulus for follow-on investigations with additional cohorts.

 The authors should describe how they corrected their measures of significance for multiple outcomes, since they are looking at a very large set of measures.

 The authors speak of the possibility of an evolutionary advantage of having a larger number of GCC repeats, but the study would not appear to be able to address that issue. First, if the population of GCC repeats is in equilibrium, then their data in Figure 1, suggests that there is a relative disadvantage of having a larger repeat (ext. < 10% with alleles > 24 repeats), with any net drive (instability) to larger repeats counterbalance by some disadvantage(s). Second, whether or not there is some advantage to the individual of having the larger number of repeats, how might that translate to increased fitness?

Is the work clearly and accurately presented and does it cite the current literature?

Yes

If applicable, is the statistical analysis and its interpretation appropriate?

Partly

Are all the source data underlying the results available to ensure full reproducibility?

Yes

Is the study design appropriate and is the work technically sound?

Partly

Are the conclusions drawn adequately supported by the results?

Partly

Are sufficient details of methods and analysis provided to allow replication by others?

Partly

Reviewer Expertise:

repeat-associated neurodevelopmental and neurodegenerative disorders

I confirm that I have read this submission and believe that I have an appropriate level of expertise to confirm that it is of an acceptable scientific standard, however I have significant reservations, as outlined above.

# Response

**Published:** 2024-09-16

**Authors:** Yasmin Iles-Caven

**Affiliations:** Bristol Medical School: Population Health Sciences, University of Bristol, Bristol, UK

The authors presented an analysis of the relationship between the number of GCC repeats in the3 range <60 and various neurocognitive and behavioral phenotypes, largely developed through surveys. The authors had speculated that the apparent instability in mother-to-son transmission, with increasing GCC repeat number in the offspring, might reflect an evolutionary advantage. However, they saw no cognitive benefit, and noted that the larger repeats were associated with several disadvantageous neurocognitive traits. The results are intriguing, although the basis for the observed associations is not known. This study should provide a stimulus for follow-on investigations with additional cohorts.

**Response:** **Thank you for pointing out the way in which these results should provide a stimulus for analyses using other cohort studies. As we have now made clearer, this is likely to be the only way in which statistical certainty can be achieved.**

 The authors should describe how they corrected their measures of significance for multiple outcomes, since they are looking at a very large set of measures.

**Response: We have added a paragraph in the introduction to emphasise that the study is expected to find pleiotropic effects rather than a single outcome. This makes it difficult to choose a sensible cut-point for a P value cut-point such as Bonferroni, which is basically concentrated on a single outcome. The most feasible way of strengthening the findings is by testing them in other large populations We have emphasised that in the revised Abstract and in the Conclusions.**

 The authors speak of the possibility of an evolutionary advantage of having a larger number of GCC repeats, but the study would not appear to be able to address that issue. First, if the population of GCC repeats is in equilibrium, then their data in Figure 1, suggests that there is a relative disadvantage of having a larger repeat (ext. < 10% with alleles > 24 repeats), with any net drive (instability) to larger repeats counterbalance by some disadvantage(s). Second, whether or not there is some advantage to the individual of having the larger number of repeats, how might that translate to increased fitness?

**Response: The possibility of an evolutionary advantage was suggested from the observations, in other studies, that the inheritance of the X chromosome from mother to son tended to be accompanied by an increase in the number of FRAXE repeats. This raised the question as to why that happened – and the hypothesis that the increase might convey some evolutionary advantage. We investigated this hypothesis with respect to neurocognitive traits and observed no sign of an advantage, but rather a decided disadvantage. We have not, at this stage, tested the hypothesis in other areas such as biological markers. This is mentioned in the Discussion section.**
